# Italy SimSmoke: the effect of tobacco control policies on smoking prevalence and smoking attributable deaths in Italy

**DOI:** 10.1186/1471-2458-12-709

**Published:** 2012-08-29

**Authors:** David Levy, Silvano Gallus, Kenneth Blackman, Giulia Carreras, Carlo La Vecchia, Giuseppe Gorini

**Affiliations:** 1 Lombardi Cancer Center at Georgetown University, Washington, DC, USA; 2 Department of Epidemiology, Istituto di Ricerche Farmacologiche Mario Negri, Via La Masa, 19-20156, Milan, Italy; 3 Pacific Institute for Research and Evaluation, Calverton, MD, 20705, USA; 4 Environmental & Occupational Epidemiology Unit, Cancer Prevention & Research Institute (ISPO), Florence, Italy; 5 Department of Clinical Sciences and Community Health, Università degli Studi di Milano, Milan, Italy

**Keywords:** Smoking prevalence, Simulation models, Tobacco control policy, Smoking attributable mortality, Italy

## Abstract

**Background:**

While Italy has implemented some tobacco control policies over the last few decades, which resulted in a decreased smoking prevalence, there is still considerable scope to strengthen tobacco control policies consistent with the World Health Organization (WHO) policy guidelines. The present study aims to evaluate the effect of past and project the effect of future tobacco control policies on smoking prevalence and associated premature mortality in Italy.

**Methods:**

To assess, individually and in combination, the effect of seven types of policies, we used the *SimSmoke* simulation model of tobacco control policy. The model uses population, smoking rates and tobacco control policy data for Italy.

**Results:**

Significant reductions of smoking prevalence and premature mortality can be achieved through tobacco price increases, high intensity media campaigns, comprehensive cessation treatment program, strong health warnings, stricter smoke-free air regulations and advertising bans, and youth access laws. With a comprehensive approach, the smoking prevalence can be decreased by as much as 12% soon after the policies are in place, increasing to a 30% reduction in the next twenty years and a 34% reduction by 30 years in 2040. Without effective tobacco control policies, a total of almost 300 thousand lives will be prematurely lost due to smoking by the year 2040.

**Conclusion:**

Besides presenting the benefits of a comprehensive tobacco control strategy, the model helps identify information gaps in surveillance and evaluation schemes that will promote the effectiveness of future tobacco control policy in Italy.

## Background

The Framework Convention on Tobacco Control (FCTC), the first treaty negotiated under the auspices of the World Health Organization (WHO), was developed in response to the globalization of the tobacco epidemic. The FCTC suggestions have been formalized in the WHO MPOWER package. In defining a set of policies, WHO suggests that each nation impose taxes on cigarettes that constitute at least 75% of the retail price, require large, bold and graphic health warnings, provide broad access to cessation treatments, conduct a well-funded mass-media campaign, and implement and enforce comprehensive smoke-free indoor air laws and advertising/marketing restrictions [1,2].

These recommendations are the result of a large and growing number of scientific studies conducted over the last five decades, showing the effectiveness of the above mentioned strategies to control tobacco. Substantial evidence indicates that higher cigarette taxes, clean air laws, advertising bans, and media campaigns can appreciably reduce adult smoking rates, especially when combined as a comprehensive strategy [3-6]. Evidence is mounting for health warnings and cessation treatment coverage. These policies not only reduce smoking initiation, but also lead current smokers to quit. Quitting can halt or even reverse many of the health problems associated with smoking [7,8]. Therefore, smoking rates, and consequently smoking attributable deaths (SAD), are reduced in response to changes in tobacco control policies.

Various countries stand in different stages of the tobacco epidemic [9] and have already introduced different more or less comprehensive and observed strategies to control tobacco [10]. Thus, each country has different benefits in introducing a new policy or in extending an already implemented anti-tobacco regulation. It is therefore important to analyze and quantify the effects of introducing new policies on smoking rates in each country. Since the ability of purely statistical studies to analyze the issue is limited, simulation models provide a useful tool in combining information from different sources to examine how the effects of public policies unfold over time in complex social systems [11,12]. One of these simulation models, the *SimSmoke* model, which has been validated in several countries [13,14] and states [13,15,16], simultaneously considers a broad array of public policies [17].

This study uses a modified version of the *SimSmoke* tobacco control policy simulation model (*Italy SimSmoke*) to examine the effect that the introduction of tobacco control policies might have on smoking rates and deaths due to smoking in Italy, which ratified the FCTC in July 2008. *Italy SimSmoke* assesses the effect of tobacco control policies, including price increases on cigarettes, smoke-free air laws, media campaigns, advertising bans, health warnings, cessation treatment policies, and youth access enforcement, on smoking rates and SADs, overall and by age and gender.

## Methods

*SimSmoke* includes a population model, a smoking model, a SAD model, and policy modules [12,18,19]. The simulation model begins in a baseline year with the population divided into smokers, never smokers, and former smokers by age and gender. The baseline year is 1999 based on the availability of a large scale study for that year and stability of policies during that period.

A discrete time, first-order Markov process is employed to project future population growth through fertility and deaths, and smoking rates through smoking initiation, cessation, and relapse. The data used in *Italy SimSmoke* are summarized in Table 1.

**Table 1:** **Data used in Italy *SimSmoke***

**Input**	**Source**	**Specifications**
**I. Population**		
**A. Population**	Institute of Health Information and Statistics of Italy (ISTAT)	Breakdowns by age and gender groups
**B. Fertility rates**	ISTAT	Breakdowns by age and gender groups
**C. Mortality rates**	ISTAT	Breakdowns by age and gender groups
**II. Smoking and attributable deaths**		
**A. Baseline smoking rates**	Multipurpose Surveys “Aspects of daily living”, carried out from 1993 onwards by ISTAT	Breakdown of current, former and never smokers by age and gender groups.
**B. Initiation rates**	Change in smoking rates between contiguous age groups	Breakdowns by age and gender groups.
**C. First year cessation rates**	Multipurpose Surveys “Aspects of daily living”	Breakdowns by age and gender groups.
**D. Relapse rates**	USDHHS (1989) and other studies	Breakdowns by age
**E. Relative risks of current and ex-smokers**	Cancer Prevention Study II (NCI 1997)	Breakdowns by age and gender.
**III. Policies**		
**A. Taxes**	Eurostat, and MPOWER Reports (2008. 2010)	Prices and taxes
**B. Clean air laws**	Past studies, MPOWER Report (2008, 2010), and labor force participation rates	Types of laws (worksite, restaurant, and other places) and their stringency
**C. Media & other educational campaigns**	Tobacco control staff in Italy and MPOWER Reports (2008. 2010)	Classification based on expenditures per capita and audience
**D. Marketing bans**	Past studies, and MPOWER Reports (2008. 2010)	Extent of bans
**E. Warning Labels**	Past studies, and MPOWER Reports (2008. 2010)	Strictness of labels
**F. Cessation Treatment Policies**	Past studies, and MPOWER Reports (2008. 2010)	Financial reimbursement, quitlines, and brief interventions
**G. Youth access**	Past studies, and MPOWER Reports (2008. 2010)	Enforcement checks, penalties, community campaigns, self-service and vending machine bans

### Population model

The population evolves over time due to deaths and births. Sex and age-specific population data for the year 1999 were available from the Italian Institute of Statistics (http://www.istat.it). Since the data were presented by age group, the data were smoothed to obtain individual ages. Mortality by age and gender and fertility rates by age come from ISTAT for the year 2005. Projections from the model keep population roughly constant, consistent with data and projected population increases from ISTAT.

### Smoking model

Within the smoking model, individuals are classified as never smokers from birth until they initiate smoking or die. They may evolve from current to former smokers through cessation or may return to current smokers through relapse. The extent of relapse depends on the number of years since quitting.

Smoking prevalence data for the period 1999–2009 were available from the Multipurpose Surveys “Aspects of daily living”, carried out from 1993 onwards by the Institute of Health Information and Statistics of Italy (ISTAT) [20]. These surveys were carried out almost every year on representative samples of the Italian population (about 24,000 families and 54,000 persons distributed in about 850 Italian municipalities of different population sizes). Smoking status was based on participant self-report as never, former, or current smoker [20]. Smoking prevalence and initiation rate data were linearly interpolated to obtain data for the missing years. Figures on the subdivision of former smokers by duration since quitting were derived from the ISTAT Multipurpose Surveys “Aspects of daily living” [20]. Because former-smokers were not distinguished by years-quit data after the first year, we used data from the Netherlands to estimate the proportions in the years-quit categories of 1 year or more.

Due to empirical challenges in measuring initiation and cessation and in order to ensure stability and internal consistency of the model, initiation rates at each age were measured as the difference between the smoking rate at that age year and the rate at the previous age year. We examined data from ISTAT regarding when smokers and former smokers stated that they first started smoking. Based on that information, we allowed initiation up through age 30.

To measure the annual cessation rate, we used data available for those who quit in the last year. Using the baseline smoking prevalence data, we constructed a cessation rate by age and gender, measured as those who quit in the last year as percent of “those currently smoking plus those who quit in the last year”. Since that rate does not allow for relapse of those who quit less than one year ago, we applied a 50% relapse rate to that measure. These rates are consistent with estimates suggested by West [21,22] and those found in studies of quitting behaviors in the Netherlands [23-25]. Since data were not available for Italy, we used U.S. relapse rates [8,26-29], but we compared ex-smoker rates and made adjustments accordingly to calibrate the model. After calibrating the model, we increased the relapse rate to 75% for ages 30–34, 70% for ages 35–44, 65% for ages 45–54, and 60% for ages 55–64 years.

### Smoking-Attributable Deaths

SADs are based on the excess risks of current and former smokers relative to never smokers. Death rates by age, gender and smoking status were calculated from death rates, smoking rates, and relative risks. The number of current and former smokers at each age was multiplied by their respective excess risk and summed to obtain total SADs.

Large scale studies of the relative risk of smoking are not available for Italy. Since Italy has a similar smoking history to the United States and is a high income country, we used relative risk estimates from the U.S. Cancer Prevention Study (CPS) II [28,30,31]. We note that Doll and Peto [32,33] found similar relative risks in prospective studies conducted in the UK. For ex-smokers, we allow relative risks to decline at the rate observed in U.S. studies [34], similar to those found in the British studies [32,33]. Moreover, CPS II relative risk estimates were in agreement with those found in Italian epidemiological studies for several mortality causes [35].

### Policy effects

*SimSmoke* includes a separate policy module for each of the major policies. Policy effect sizes are in terms of percentage reductions applied to smoking prevalence in the year when the policy was implemented, and, unless otherwise specified, applied to initiation and cessation rates in future years. Policies and effect sizes are summarized in Table 2. The effect of implementing a policy depends on the prior level of that policy (e.g., the incremental effect of a complete work site ban is less when a country already has a partial worksite ban than if no smoking ban is implemented). We input data on policy levels for each year from the baseline year through 2009, based on the WHO MPOWER reports [1,2] and on data provided by tobacco control staff in Italy. 

**Table 2:** Policies, description and effect sizes of the SimSmoke model

**Policy**	**Description**	Potential percentage effect*
		Through price elasticity:
***Tax Policy***	Cigarette price index	−0.3 ages 15–24
		−0.2 ages 25–34
		−0.1 ages 35 and above
***Clean Air Policies***		
**Worksite total Ban**	Ban in all areas	6% with variations by age and gender
**Worksite ban except ventilated areas**	Smoking restricted to ventilated areas in all indoor workplaces	4% with variations by age and gender
**Worksite ban limited to common area**	Smoking limited to non ventilated common area	2% with variations by age and gender
**Restaurant total ban**	Ban in all indoor restaurants in all areas	1% effect
Restaurant ban except separate areas	Ban in all restaurants except in designated areas	0.5% effect
Other places total ban	Ban in 3 of 4 (malls, retail stores, public transportation and elevators)	1% effect
**Enforcement and publicity**	Government agency is designated to enforce and publicize the laws	Effects reduced by as much as 50% if 0 enforcement
***Mass Media Campaigns***		
**Highly publicized campaign**	Campaign publicized heavily on TV (at least two months of the year) and at least some other media	3.25% effect (doubled when accompanied by local programs)
**Moderately publicized campaign**	Campaign publicized sporadically on TV and in at least some other media, and a local program	1.8% effect (doubled when accompanied by local programs)
**Low publicity campaign**	Campaign publicized only sporadically in newspaper, billboard or some other media.	0.5% effect (doubled when accompanied by local programs)
***Marketing Ban***		
**Comprehensive marketing ban**	Ban is applied television, radio, print, billboard, in-store displays, sponsorships and free samples	5% reduction in prevalence,
		6% reduction in initiation,
		3% increase in cessation rates
**Total Advertising Ban**	Ban is applied all media television, radio, print, billboard	3% reduction in prevalence,
		4% reduction in initiation,
		2% increase in cessation rates
**Weak advertising ban**	Ban is applied some of television, radio, print, billboard	1% reduction in prevalence and initiation only
**Enforcement and publicity**	Government agency is designated to enforce the laws	Effects reduced by as much as 50% if 0 enforcement
***Warning Labels***		
**Strong**	Labels are large, bold and graphic	2% reduction in prevalence
		2% reduction in initiation
		4% increase in cessation rate
**Weak**	Laws cover less than 1/3 of package, not bold or graphic	1% reduction in prevalence & initiation rates, 2% increase in cessation rates
**Publicity**	Health information is well publicized	1% additional effect on prevalence and initiation rates
***Cessation Treatment Policy***	Complete availability and reimbursement of pharmaco- and behavioral treatments, quitlines, and brief interventions	4.75% reduction in prevalence, 39% increase in cessation rate
***Youth Access Restrictions***		
**Strongly enforced & publicized**	Compliance checks are conducted regularly, penalties are heavy, and with publicity is strong, vending machine and self-service bans	30% reduction for age < 16 in prevalence and initiation only,
		20 % reduction for ages 16–17 in prevalence and initiation only
**Well enforced**	Compliance checks are conducted sporadically, penalties are potent, and little publicity	15 % reduction for age < 16 in prevalence and initiation only,
		10 % reduction for ages 16–17 in prevalence and initiation only
**Low enforcement**	Compliance checks are not conducted, penalties are weak, and no publicity	3 % reduction for age < 16 in prevalence and initiation only,
		2 % reduction for ages 16–17 in prevalence and initiation only

* Unless otherwise specified, the same percentage effect is applied as a percentage reduction in the prevalence and initiation rate and a percentage increase in the cessation rate, and is applied to all ages and both genders. The effect sizes are shown relative to the absence of any policy. They are based on literature reviews, advice of an expert panel and model validation.

*Tax policy* in *SimSmoke* uses cigarette prices adjusted for inflation as the policy input through 2010, which changes in price are translated into changes in smoking prevalence through elasticities (i.e., the percent change in smoking prevalence for a one percent change in price). The model uses the total cigarette excise tax (including ad valorem and specific) to adjust future prices. Gallus and colleagues [36] obtained price elasticities for Italy comparable to those in the U.S., with minimal tendencies for smokers to substitute to smuggled cigarettes and hand rolled cigarettes [37]. The price effects are assumed to be the same as in U.S. model, where the elasticity for smoking prevalence is −0.3 for those through age 24, -0.2 for those aged 25–34, and −0.1 for those aged 35 and above. Since the vast majority of tobacco users smoke tobacco, the price was based on the tobacco price index deflated by the consumer price index from 1999 through 2009 (stats.oecd.org/index.aspx? querytype = view&queryname = 221). We examine the effect of an increase in future tobacco prices by 25% in the first year (i.e. 2011), an additional 20% the second (relative to the preceding year), 15% the third, 10% the fourth and 5% the fifth year. This would imply a total 99.3% price increase over the next 5 years, which would be similar to increasing the current ad valorem and specific excise taxes from its current level by around 20%.

*Smoke-free air policy* considers smoking restrictions in: 1) worksites, 2) restaurants and bars, and 3) other places, with their effect dependent on enforcement and publicity (based on the level of tobacco control campaigns). With strong enforcement and publicity, the effect of a ban in restaurants is 2%, in pubs and bars is 1%, in worksites is 6%, and in other public places is 1%, with the full effects dependent on full enforcement, based on recent reviews [4,38] and studies from Italy [37,39-41]. Italy now has strong smoke-free air laws. Dating back to 1975, there were bans on smoking in many public places, including hospital wards, in all the school classes, libraries, cinemas and museums, and in public transport (http://www.salute.gov.it/stiliVita/paginaInternaMenuStiliVitaFaq.jsp?id=44&menu=strumentieservizi&label=faq), which is designated as a full ban in other public places. A ban was not implemented until 2005 for all worksites, bars and restaurants. While there is an exclusion for ventilated areas, it is very costly to comply with this regulation. Italy is considered to have weak worksite laws (ban in government and health facilities), and no ban in restaurant or pubs and bars through 2005, and then complete smoke-free air laws (worksites, bars and restaurants), and other public places since 2005. Based on information in the MPOWER Report [1] for 2007, the enforcement level is set to 8 out of 10.

MPOWER [1] distinguishes enforcement and 4 levels of *direct (advertising and point of sale) and indirect (sponsorships, branding, or promotional discounts) marketing* as: 1) none, 2) minimal (up to 3 direct or 1 indirect ban), 3) moderate (4–6 direct or 1 indirect), and 4) complete (direct and indirect). With a complete direct and indirect ban, prevalence is reduced by 5% and initiation by 6%, and cessation is increased by 3% [4,42,43]. Since 1983, there was a ban on direct advertising in newspapers, radio, and television. In 1991, the ban was extended to cover most indirect advertising, with some minor changes in 2004 related to Formula One racing sponsorship and free distribution practices. According to the MPOWER Reports, there is a ban on all types of indirect advertising. On that basis, we categorize Italy as having a complete (level 4) marketing ban from the baseline year to the present. Based on the 2009 MPOWER report [2], however, enforcement is 10 for direct and 3 for indirect, which were averaged to set enforcement to 7 out of 10 for all years.

MPOWER provides 4 levels for *health warnings* on cigarette packs: none, minimal (< 30% of the principal display area), moderate (covering at least 30% of the display area and includes seven pack warning criteria) and complete (covers at least 50% of the display area and includes all seven warning criteria and a ban on deceitful terms). Based on Levy et al. [4] and more recent studies [44-46] strong health warnings reduce prevalence by 2% and initiation by 1%, and increase cessation by 5%. When warning labels are moderate (low), prevalence is reduced by 0.75% (0.5%), cessation is increased by 2.5% (1.0%) and initiation is reduced by 0.5% (0.5%). When warning labels are moderate or high, a synergy from publicity through tobacco control campaigns reduces prevalence by an additional 1% and increases cessation by an additional 2% if tobacco control funding is high and half of that amount if it is medium. Health warnings were minimal in Italy until 2003. Following EU directives, warnings were increased to cover at least 30% of both sides of the pack in 2003, but have not included pictorial images. Thus, we assigned a moderate warning from 2003 to 2010.

*SimSmoke* specifies three levels for *tobacco control campaigns*: 1) low: a national agency and minimal funding or employees, 2) medium: a national agency plus 10+ employees or per capita expenditures over $0.10 (USD) per capita, and 3) high: a national agency and expenditures over $0.50 per capita, and incorporates synergies arising from publicity surrounding other tobacco control policies. Based on reviews [4,47-49] and several recent studies [50-53], a well-funded tobacco control campaign in conjunction with other policies yields an effect size of 6.5%. A low campaign yields an effect size of 1%. Without other policies in place, the effects are reduced by half. Anti-tobacco media campaigns have been implemented in Italy in 2002–2003, 2004, 2005, 2009, and 2010 (http://www.salute.gov.it/servizio/campagna.jsp). The 2010 media campaign was a 35 second television spot delivered on 15 days in summer, and cinema and radio spots delivered from November 15 to December 15, 2010. The MPOWER Report [1,2] indicates health expenditures of less than $1 USD per person. Because of the sporadic and generally low level of he campaign, we assign a low intensity media campaign to Italy since 1999.

A strongly enforced policy *restricting the purchase of cigarettes by youth* and with bans on self-service and vending machines reduces smoking prevalence by those under the age of 18 by as much as 25% [54]. While retailers have a duty to ensure tobacco products are not sold to anyone under the age of 16, youth are rarely denied purchase of cigarettes in Italy [55]. Enforcement is set to a zero level since the baseline year of the model, with no bans on vending machines or self-service displays.

*Cessation treatment* includes four sub-policies. Treatment coverage is based on the places providing cessation treatments (physician offices, hospitals, community centres, provider offices and other). With a high-level media campaign, prevalence is reduced by 2.25%, and the cessation rate is increased by 12% in future years [48,56]. The effect of a quitline also depends on publicity, with prevalence reduced by 0.5% and cessation increased by 5% in all future years [56]. An index for pharmacotherapy availability was developed, which was given full weight if: i) both nicotine replacement therapy (NRT) and buproprion were available, and ii) NRT is available in a pharmacy without prescription or general store. When fully implemented, prevalence is reduced by 1.0%, and the cessation rate is increased by 6% in all future years [57,58]. Brief interventions would involve at minimum that health care providers advise and assist in cessation. When fully implemented, prevalence is reduced by 0.5% in the first year, and the cessation rate is increased by 10%, based on evidence provided in Levy et al. [4]. When all sub-policies are implemented (from a scenario of no policy), smoking prevalence is reduced by 4.75% and the cessation rate is increased by 39.3%.

Since 2003, NRT has been available OTC in pharmacies, and was only provided by prescription in previous years. Buproprion is available with a prescription for all years. The National Health Service provides no reimbursement to smokers for pharmacotherapy or behavioral treatments [59] and there is little use, although heavy smokers are more frequent compared with other high income nations [60,61]. Several Smoking Cessation Services are not adequately funded and their activities are only periodically implemented [62]. According to the MPOWER Report [2] and based on the characteristics described above, financial coverage of treatments is provided only in some places for primary care facilities, hospitals, and offices of health professionals, and we assume those values for all years. MPOWER [2] indicates that there is a quitline, which Italian sources indicate has been in existence since 1999, but is available for limited hours and serves only about 0.6-0.7% of smokers [61], and is considered to be passive. A recent survey found that only 22% of smokers reported having only received advice to quit by their general practitioners [63], but another recent survey **(**PASSI; see http://www.epicentro.iss.it/passi/english.asp) found that health professionals delivered smoking cessation counseling to 60% of smokers. We set brief interventions at a level of 30% for all years.

### The model outcomes

As described above, the model estimates the effects over time for two primary outcomes: smoking prevalence and SADs. Smoking prevalence is provided for the population aged 15 and above, but the model also has the capability to provide breakdowns by age. Separate results are provided for males and females and overall. The model estimates these outcomes for the tracking period, which is from 1999 to 2010, and projects future outcomes for 2011 through 2040.

To calibrate the model, we compared trends in the model over the period from 1999 to 2002 to data gleaned from ISTAT surveys over the period 1993–2002. To validate the model, we compared trends from the model to data from ISTAT for more recent years.

In examining the potential effect of future policies consistent with the FCTC, we first present the status quo case, where tobacco control policies are maintained at their 2010 level. We then consider the effect of varying levels of tobacco control policies in isolation and through a comprehensive tobacco control strategy involving all policies being simultaneously implemented in 2011. In comparing the effect of policies to the status quo, we focus on the relative change in smoking prevalence, i.e., the change in smoking prevalence from the status quo in response to the future policy scenario divided by the status quo smoking prevalence. For SADs, we calculate lives saved as the difference between the number of deaths under the new policy and the number of deaths under the status quo.

For the present study, no ethical approval is required since only aggregate data obtained from published databases were used

## Results

### Predictions of smoking prevalence from 2002 to 2010

Smoking prevalence is reported as a percent of the population aged 15 and above. Between 1999 and 2010, the model predicts that male smoking rates fell from 31.8% to 26.8%, a 15.7% decline in relative terms, and that female smoking rates decreased from 16.7% to 14.9%, an 11% decline in relative terms. Comparing to ISTAT survey data for 1999 and 2003, male smoking prevalence fell by 4.3% by 2003 compared to 2.6% predicted by the model, and by 2008 falling by 11.7% according to surveys compared to 13.9% predicted by the model. For females, the decrease of 1.8% is greater than the 0.6% predicted decrease by the model for 2003, and for 2008 the surveys show a 4.7% actual decrease compared to a 10.2% decrease predicted by the model. The survey results for females were highly sensitive to the year chosen, but the model predicts better relative to 2002. The model also over-predicts reductions at the younger (<25 years old) and increases at the older (above 45 years old) ages, but is more accurate for the 25–44 year old age group, the less volatile one.

### Role of policies implemented in 2010 in reducing future smoking prevalence and deaths

The estimates of smoking prevalence under the *status quo* and under varying policy scenarios are shown in Table 3 and 4 for males and females respectively. The total number of projected SADs and lives saved for each year and the summed numbers over the years 2011–2040 is displayed for the different policies in Table 5. and 6 for males and females, respectively.

**Table 3:** SimSmoke Projections: Male smoking prevalence for ages 15 and above, Italy, 2010-2040

***POLICY/YEAR***	***2010***	***2011***	***2020***	***2030***	***2040***
***Status Quo Policies***	26.8%	26.6%	24.3%	22.2%	20.4%
***Independent Policy Effects***					
Rolling Price Changes**	26.8%	25.8%	22.0%	19.6%	17.6%
*Complete Smoke Free Air Laws*	26.8%	26.5%	24.3%	22.2%	20.4%
*Comprehensive Marketing Ban*	26.8%	26.4%	24.1%	22.0%	20.2%
*High Intensity Tobacco Control Campaign*	26.8%	24.9%	22.6%	20.4%	18.6%
*Strong Health Warnings*	26.8%	26.5%	24.2%	22.0%	20.2%
*Strong Youth Access Enforcement*	26.8%	26.5%	23.8%	21.2%	19.1%
*Cessation Treatment Policies*	26.8%	26.1%	23.2%	20.9%	19.1%
***Combined Policy Effects***					
*All above*	26.8%	23.4%	18.4%	15.5%	13.3%
% Change in Smoking Prevalence from Status Quo*					
***Independent Policy Effects***					
Rolling Price Changes**		−2.7%	−9.6%	−11.6%	−13.5%
*Complete Smoke Free Air Laws*		−0.2%	−0.2%	−0.1%	−0.1%
*Comprehensive Marketing Ban*		−0.7%	−0.8%	−0.9%	−1.0%
*High Intensity Tobacco Control Campaign*		−6.1%	−7.2%	−8.1%	−8.7%
*Strong Health Warnings*		−0.2%	−0.7%	−1.0%	−1.2%
*Strong Youth Access Enforcement*		−0.4%	−2.3%	−4.3%	−6.2%
*Cessation Treatment Policies*		−1.9%	−4.6%	−5.9%	−6.5%
***Combined Policy Effects***					
*All above*		−12.0%	−24.5%	−30.3%	−34.6%

* Measured relative to the status quo in the same year, i.e., (SRp,t-SRStatus quo,t)/ SRStatus quo,t, where SRp,t is the smoking rate in year t with policy p and SRStatus quo,t is the smoking rate in year t under the status quo.

** 25 % change 2010–2011, 20 % change 2011–2012, 15 % change 2012–2013, 10 % change 2013–2014, 5 % change 2014–2015. Prices kept at 2015 level throughout the remaining projection period.

**Table 4:** SimSmoke Projections: Female smoking prevalence for ages 15 and above, Italy, 2010-2040

***POLICIES***	***2010***	***2011***	***2020***	***2030***	***2040***
***Status Quo Policies***	14.9%	14.8%	14.1%	13.2%	12.0%
***Independent Policy Effects***					
Rolling Price Changes**	14.9%	14.4%	12.8%	11.7%	10.5%
*Complete Smoke Free Air Laws*	14.9%	14.8%	14.1%	13.2%	12.0%
*Comprehensive Marketing Ban*	14.9%	14.7%	14.0%	13.1%	11.9%
*High Intensity Tobacco Control Campaign*	14.9%	13.9%	13.1%	12.1%	11.0%
*Strong Health Warnings*	14.9%	14.8%	14.0%	13.1%	11.9%
*Strong Youth Access Enforcement*	14.9%	14.8%	13.8%	12.6%	11.3%
*Cessation Treatment Policies*	14.9%	14.5%	13.5%	12.4%	11.3%
***Combined Policy Effects***					
*All above*	14.9%	13.0%	10.8%	9.4%	8.0%
Relative Change in Smoking Prevalence from Status Quo*					
***Independent Policy Effects***					
Rolling Price Changes**		−2.7%	−9.2%	−10.9%	−12.6%
*Complete Smoke Free Air Laws*		−0.2%	−0.2%	−0.2%	−0.1%
*Comprehensive Marketing Ban*		−0.7%	−0.8%	−0.9%	−0.9%
*High Intensity Tobacco Control Campaign*		−6.2%	−7.2%	−7.9%	−8.5%
*Strong Health Warnings*		−0.2%	−0.7%	−0.9%	−1.1%
*Strong Youth Access Enforcement*		−0.3%	−2.2%	−4.1%	−6.1%
*Cessation Treatment Policies*		−1.9%	−4.4%	−5.7%	−6.4%
***Combined Policy Effects***					
*All above*		−12.0%	−23.5%	−28.9%	−33.2%

* Measured relative to the status quo in the same year, i.e., (SRp,t-SRStatus quo,t)/ SRStatus quo,t, where SRp,t is the smoking rate in year t with policy p and SRStatus quo,t is the smoking rate in year t under the status quo.

** 25% change 2010–2011, 20% change 2011–2012, 15% change 2012–2013, 10 % change 2013–2014, 5% change 2014–2015. Prices kept at 2015 level throughout the remaining projection period.

**Table 5:** Male smoking attributable deaths, SimSmoke Italy, 2010-2040

***Policies***	**2010**	**2020**	**2030**	**2040**	**Cumulative**
***Status Quo Policies***	60,652	64,855	61,949	54,607	**1,854,650**
***Independent Policy Effects***					
Rolling Price Changes**	60,652	63,424	58,657	51,042	**1,788,696**
*Complete Smoke Free Air Laws*	60,652	64,805	61,837	54,501	**1,852,354**
*Comprehensive Marketing Ban*	60,652	64,697	61,598	54,261	**1,847,374**
*High Intensity Tobacco Control Campaign*	60,652	63,428	58,619	51,128	**1,785,387**
*Strong Health Warnings*	60,652	64,731	61,592	54,138	**1,847,242**
*Strong Youth Access Enforcement*	60,652	64,855	61,937	54,493	**1,854,008**
*Cessation Treatment Policies*	60,652	63,939	59,387	51,356	**1,801,675**
***Combined Policy Effects***					
*All above*	60,652	60,876	52,195	43,617	**1,654,086**
***Absolute Change in Attributable Deaths from Status Quo***					**2011-2040**
***Independent Policy Effects***					
Rolling Price Changes**		1,431	3,292	3,565	**65,953**
*Complete Smoke Free Air Laws*		50	112	105	**2,296**
*Comprehensive Marketing Ban*		158	351	345	**7,276**
*High Intensity Tobacco Control Campaign*		1,427	3,330	3,479	**69,263**
*Strong Health Warnings*		124	357	469	**7,407**
*Strong Youth Access Enforcement*		0	12	114	**641**
*Cessation Treatment Policies*		916	2,562	3,250	**52,975**
***Combined Policies***		3,979	9,754	10,990	**200,563**

** 25% change 2010–2011, 20% change 2011–2012, 15% change 2012–2013, 10 % change 2013–2014, 5% change 2014–2015. Prices kept at 2015 level throughout the remaining projection period.

**Table 6:** Female smoking attributable deaths, SimSmoke Italy, 2010-2040

***POLICIES***	**2010**	**2020**	**2030**	**2040**	**Cumulative**
***Status Quo Policies***	18,433	24,390	28,632	29,488	**783,587**
***Independent Policy Effects***					
Rolling Price Changes**	18,433	23,816	27,102	27,695	**753,057**
*Complete Smoke Free Air Laws*	18,433	24,370	28,578	29,429	**782,486**
*Comprehensive Marketing Ban*	18,433	24,326	28,465	29,305	**780,155**
*High Intensity Tobacco Control Campaign*	18,433	23,816	27,072	27,670	**751,293**
*Strong Health Warnings*	18,433	24,348	28,483	29,260	**780,432**
*Strong Youth Access Enforcement*	18,433	24,390	28,629	29,459	**783,432**
*Cessation Treatment Policies*	18,433	24,076	27,546	27,876	**760,709**
***Combined Policy Effects***					
*All above*	18,433	22,849	24,189	23,890	**692,046**
***Absolute Change in Attributable Deaths from Status Quo***					**2011-2040**
***Independent Policy Effects***					
Rolling Price Changes**		574	1,530	1,792	**30,530**
*Complete Smoke Free Air Laws*		21	54	59	**1,100**
*Comprehensive Marketing Ban*		65	167	182	**3,432**
*High Intensity Tobacco Control Campaign*		575	1,560	1,818	**32,294**
*Strong Health Warnings*		42	149	227	**3,155**
*Strong Youth Access Enforcement*		0	3	29	**155**
*Cessation Treatment Policies*		314	1,085	1,611	**22,877**
***Combined Policies***		1,541	4,442	5,598	**91,541**

** 25% change 2010–2011, 20% change 2011–2012, 15% change 2012–2013, 10% change 2013–2014, 5% change 2014–2015. Prices kept at 2015 level throughout the remaining projection period.

#### Status quo Scenario

If tobacco control policies remain unchanged from their 2010 levels, as in the *status quo* scenario, male adult smoking is projected to decrease in absolute terms by 2.5 percentage points (9.2% in relative terms) from 26.8% to 24.3% over the 10 years between 2010 and 2020, by 4.6 percentage points to 22.2% by 2030, and by 6.4 percentage points (23.8% in relative terms) to 20.4% over a 30-year projection to 2040. Female smoking prevalence is projected to decrease by 0.8% in absolute terms (5.2% in relative terms) in 10 years, by 1.7% (11.4% in relative terms) in 20 years, by 2.8% (19.1% in relative trms) over the period 2010–2040.

The estimated number of SADs in 2010 is 60,652 for males and 18,433 for females. The projected number of male SADs in Italy is projected to rise through 2020 and fall below 2010 levels in 2033. Relative to 2010, male deaths are projected to increase by 4,203 over the 10-year period to 2020 and by 1,297 over the 20-year period. However, male deaths per year decreased by 6,045 over the 30-year period to 2040. Female SADs are initially 18,433 and projected to increase by 5,957 over a 10-year horizon, by 10,198 over a 20-year period, and by 11,054 over a 30-year period.

#### The effects of individual MPOWER policies

Relative to the status quo scenario (the percentage change from the status quo), smoking rates are projected to decline by 2.7% for both males and females by 2011 with an increase in specific taxes which produces a 25% increase in the retail price. By the end of a 30-year projection period, the year 2040, the male smoking prevalence is projected to decline by about 13.5% and the female smoking prevalence is predicted to decline by 12.6% from the rolling price increases from 2011 to 2015. Youth smoking prevalence declines at a greater rate as a result of tax increases than adult prevalence in the model, and is the primary reason that taxes continue to reduce adult smoking rates over time. The tax increases slow the growth of deaths, with 5,357 lives saved (3,565 male and 1,792 female) in the year 2040. Summing over years from 2011 (the first year that deaths are averted) through 2040, 96,483 deaths are averted by 2040 with the effects still growing.

Comprehensive smoke-free air laws are already largely in place, and only require stronger enforcement. They are predicted to lead to a 0.1% reduction in smoking prevalence by 2040 relative to the *status quo* scenario in 2010. Similarly, comprehensive advertising restrictions are in place, and need better enforcement. The model predicts a 0.7% immediate reduction in smoking prevalence after one year, increasing to about a 1.0% reduction by 2040.

For a well-funded and publicized campaign that is sustained over time, the model predicts a 6.1% immediate reduction in smoking prevalence after one year, changing to an 8.5% reduction by 2040. There is a projected 5,297 fewer smoking-attributable deaths in 2040, cumulating to a total of 101,557 lives saved from 2011 to 2040. The effect of a warning that covers at least 50% of the principal display area of the pack and includes all seven pack warning criteria is projected to yield a 0.2% reduction in smoking prevalence after one year for both males and females, increasing to about a 1.1% reduction by 2040. Health warnings are projected to avert 696 fewer SADs in the year 2040 alone, with 10,562 deaths averted in total by 2040.

Enforcement of youth access laws is predicted to have a small immediate effect (0.4%) increasing to 6.2% by 2040. Youth access laws are projected to prevent 143 smoking attributable deaths in 2040, with a total of 796 lives saved over the period 2010–2040.

Comprehensive smoking cessation treatment policies are projected to reduce adult smoking prevalence by 4% in 2020. These relative reductions increase to 6.5% in 2040. The policies are projected to avert 4,861SADs in the year 2040, with a total of 75,852 deaths averted in total by 2040.

The final scenario projects the effect for a combination of all of the policies above. The model predicts an immediate 12.0% reduction in smoking prevalence in the first year. By 2040, this smoking prevalence is projected to decrease by 34.6% in males and 33.2% in females. Under these same policies, the model projects 16,588 less annual SADs in 2040. The cumulative number of deaths averted by 2040 is 292,104, of which 200,563 are males and 91,541 females.

**Figure 1:**
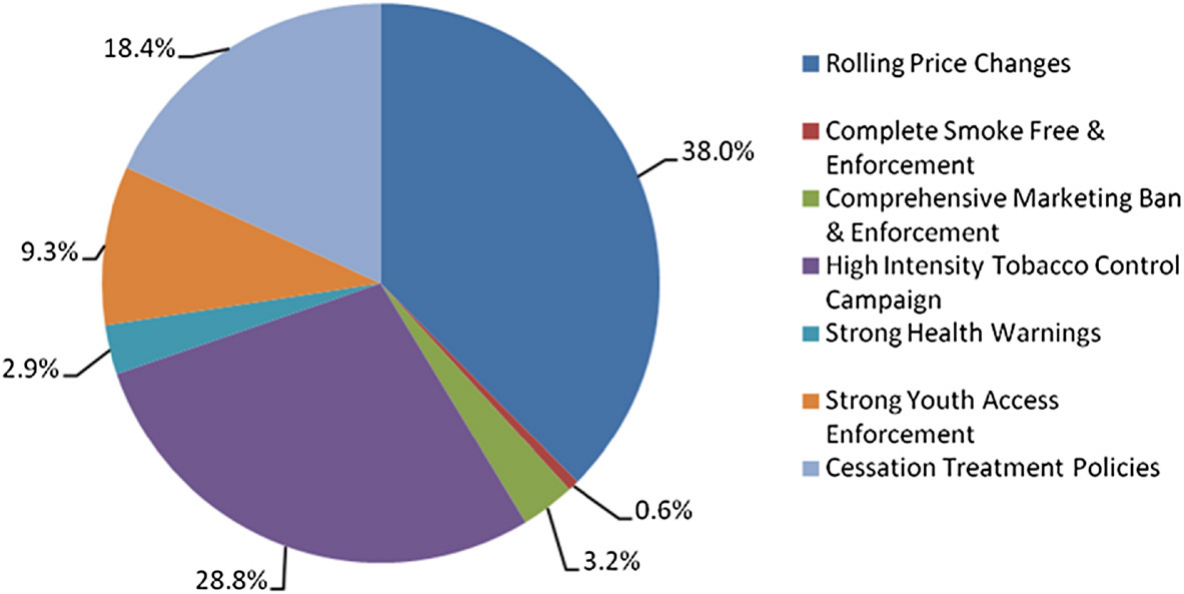
Contribution of each of the policies as a percent of the predicted 24.5% reduction in male smoking prevalence in 2020.

Figure 1 shows the relative contribution of policies as part of a comprehensive package in 2020. Of the reduction in male smoking prevalence in 2020 obtained through a comprehensive set of policies, 38% is due to progressively doubling retail prices of cigarettes, while 29% is from a media campaign, 18% from a comprehensive cessation treatment program, and less than 10% is due to youth access enforcement. By 2040, the relative contribution of price decreases slightly along with media campaigns, while the effect of youth access and health warning increases. Youth enforcement and taxes tend to have less immediate effects on mortality than the other policies, since they have their largest effects on youth.

## Discussion

*Italy SimSmoke* applies population, smoking prevalence, and policy data for that nation and modified parameter values to the established *SimSmoke* model. The model's credibility is supported by validation in countries with sufficient data to confirm predicted trends. While Italy has implemented some tobacco control policies over the last decade (but not over the last few years [10]), there is still considerable scope to strengthen tobacco control policies consistent with the MPOWER policy guidelines. Using the *SimSmoke* model, we have presented a short and long-term projection of the role of various tobacco control policies in reducing smoking prevalence and, subsequently, the number of SADs. The smoking prevalence can be decreased by as much as 12% in the first year, and by approximately 34% in 30 years.

Because of the natural progression of most of tobacco-related illnesses, reductions in smoking prevalence have a relatively small impact on the number of SADs in the short-term. However, by 2040, more than 18,000 deaths can be averted in that year alone with the stronger set of policies. Without effective tobacco control policies, 330,000 lives will be lost due to smoking by the year 2040.

We recommend interpreting these projections in a conservative manner. The model's results depend on the reliability of data, and of the estimated parameters and assumptions used in the models.

The smoking prevalence findings depend first on estimates of the rates of smoking in 1999, and initiation, cessation and relapse rates. The model over-predicted the reduction in younger smokers and under predicted the reduction in older smokers. Future smoking rates will depend especially on rates of younger smokers, and are thus subject to uncertainty especially for predictions further into the future. Reliable data were not available for relapse rates except for the US, and are based on US rates [8,26-28]. Data on cessation rates or ex-smokers by years quit were not available for Italy, so rates from the Netherlands were used. It will be important to monitor cessation rates or the distribution of ex-smokers by years quit in future years to gauge the impact of tobacco control policies. When more data becomes available on smoking prevalence, the model can be validated to see how well it has predicted trends in recent years. In addition, data on policies in effects should be collected over time, so that data can be used in updates of the model and in policy evaluations.

The estimated relative risks for total mortality of smokers are based on CPS-II from the US [8,28,31]. However, relative risk estimates by cause of death from Italian epidemiological studies are in agreement with the CPS-II [35]. Previous SADs estimates for Italian men [35] using SAMMEC and Peto’s method [64] showed a peak in SADs in 1985, and then a decrease. Our results instead peak after 2020. Both SAMMEC and Peto’s methods use relative risks of mortality for about 20 tobacco-related diseases from the CPS II study, and mortality figures for these specific causes. In *Italy SimSmoke* we used the relative risks of dying for smokers and former smokers from the CPS II study, and we applied these risks to the overall mortality. The differences in the methods for calculation may explain the differences between our SADs estimates and those from SAMMEC and Peto’s methods. At least part of the discrepancies in SADs is due to the fact that total mortality estimates used in SimSmoke include causes of death not included when calculating mortality by cause. Notably, the projections do not include the additional deaths averted due to reductions in second hand smoke exposure. In countries with a high number of male smokers and a low number of female smokers, a large number of female non-smokers are exposed to smoke in the home. Recent data showed that, although the Italian smoking ban introduced in 2005 substantially decreased SHS exposure, the prevalence of SHS exposure for non-smokers was 10.2% in public places, 15.6% at home, and 17.9% in transport [65].

The policy effect sizes depend on underlying assumptions, estimated parameters of the predicted effect on initiation and cessation, and assumptions about the interdependence of policies. Knowledge of the different effects of each policy varies [4]. For example, many studies, with relatively consistent results, have been done of the effects of price. There are also many studies of clean air laws, with results somewhat less consistent than those of prices/taxes, but still falling into similar ranges. Studies on media/tobacco control campaigns and advertising bans provide a broad range of estimates. Information on the effect of health warnings and cessation treatment policies is generally lacking. Better understanding of the interactive effects of policies is also needed. We have made the conservative assumption that the effects of each policy are a constant proportion of the smoking rate independent of other policies. Evidence indicates that public policies may be synergistic [6] through their cumulative impact on social norms and their reinforcing effects on smokers’ motivation to quit. Furthermore, due to a lack of studies, we were not able to incorporate how the tobacco industry would respond or adapt to changes in tobacco control policies via the development of innovative tobacco marketing and novel tobacco products, such as smokeless tobacco, and cigars. In addition, we were not able to explicitly incorporate network effects through the workplace, peers and parents.

## Conclusion

*SimSmoke* results highlight the relative contribution of numerous policies to reducing the tobacco health burden. When the tax increases by large percentages, stronger clean air and youth access laws are implemented, publicized and enforced, a strict advertising and marketing law is promulgated and enforced, strong warning labels are introduced, a high publicity media campaign is coordinated with the other policies and a strong comprehensive cessation treatment program is implemented, the smoking rate is projected to fall by 34.6% (33.2%) in males (females) in relative terms and 292,104 total deaths are averted by the year 2040. Tax/price increases are also likely to increase government tax revenues [66], part of which can and should be earmarked to pay for media campaigns and cessation treatment, and to enforce smoke-free-air laws and anti-tobacco marketing policies.

## Competing interest

Authors declare that there are no competing interest.

## Authors’ contributions

DTL developed the model and wrote the first draft of the manuscript in collaboration with SG and GG. KB, GC, CLV gave substantial contributions to interpretation of data; all authors critically revised the manuscript and approved its final version.

## Pre-publication history

The pre-publication history for this paper can be accessed here:

http://www.biomedcentral.com/1471-2458/12/709/prepub
